# Frontal Fibrosing Alopecia: An Observational Single-Center Study of 306 Cases

**DOI:** 10.3390/life13061344

**Published:** 2023-06-08

**Authors:** Marcos Carmona-Rodríguez, Fernando Moro-Bolado, Guillermo Romero-Aguilera, Ricardo Ruiz-Villaverde, Víctor Carriel

**Affiliations:** 1 Servicio de Dermatología, Hospital General Universitario, 13005 Ciudad Real, Spain; 2 Servicio de Dermatología, Hospital Universitario San Cecilio, 18016 Granada, Spain; 3 Instituto de Investigación Biosanitaria, ibs.GRANADA, 18016 Granada, Spain; 4 Departamento de Histología, Grupo de Ingeniería Tisular, Universidad de Granada, 18016 Granada, Spain

**Keywords:** trichology, frontal fibrosing alopecia, lichen planopilaris, hair loss, alopecia, erythema, follicular hyperkeratosis

## Abstract

(1) Background: Frontal fibrosing alopecia (FFA) is a scarring alopecia that predominantly affects postmenopausal women; (2) Methods: A retrospective, observational, single-center study was conducted in the Hospital General Universitario in Ciudad Real, Spain, including all patients diagnosed with FFA between 2010 and 2021; (3) Results: A total of 306 patients (296 women and 10 men) were included in our study. The mean age of onset was 59.5 years. The severity of this disease was evenly distributed between mild (147 patients) and severe (149 patients) forms. There was a positive, statistically significant, medium correlation between the severity of the disease and its time of progression. Moreover, hypothyroidism was present in 70 patients (22.9%) and classic signs of concomitant lichen planopilaris were observed in just 30 patients (9.8%), while other forms of lichen planus were uncommon. The estimated prevalence in our population is 0.15% and the incidence is 15.47 new cases per 100,000 inhabitants; (4) Conclusions: The time of progression was positively correlated with the severity of FFA. However, the presence of clinical signs, such as inflammatory trichoscopic signs, was not associated with the progression of this condition.

## 1. Introduction

**Figure 1:**
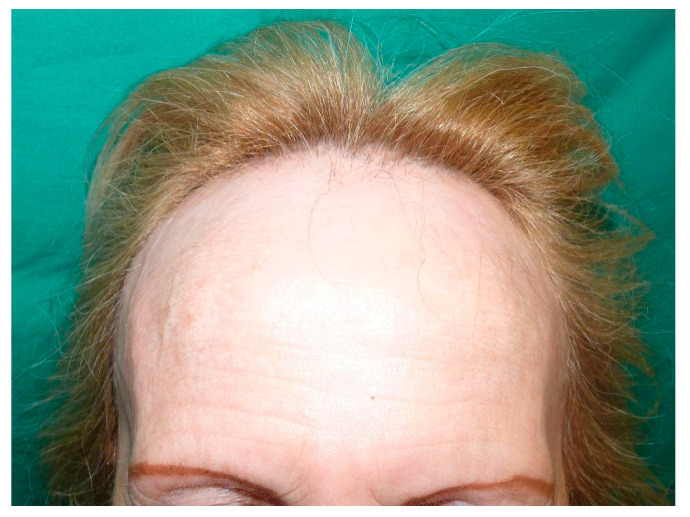
Recession of the frontotemporal hairline in a patient with severe frontal fibrosing alopecia (AFF), leaving behind a pale uniform alopecic band associated with a total eyebrow alopecia.

Frontal fibrosing alopecia (FFA) is a primary scarring and irreversible type of alopecia first described in a group of postmenopausal women by Kossard in 1994 [1]. Clinically, it causes a homogeneous, progressive and often symmetric recession of the frontotemporal hairline, leading to scarring of the uniform alopecic band (Figure 1) [1,2]. This condition frequently entails eyebrow alopecic affection, which can also precede hairline recession, eyelash alopecia or even peripheral hair loss, such as axillary, pubic or extremity hair [3]. In addition, the inflammatory infiltrate of clinically affected areas may lead to the development of erythema and/or follicular hyperkeratosis, which can cause pruritus and trichodynia [4,5].

Interestingly, FFA was initially described as a lichen planopilaris (LPP) variation by Kossard himself in 1997 [6], since the main histological findings seemed to be indistinguishable between both conditions [7]. Essentially, in both entities, there is a lymphocytic inflammatory infiltrate surrounding the upper section of hair follicles, a concentric perifollicular lamellar fibrosis and, lastly, hair destruction [1]. Although there are limited data about its prevalence, there has been a notable increase in diagnosed cases and publications regarding this entity in the last few years.

The FFA pathogenesis still remains unknown, although there seems to be hormonal factors involved, as the majority of patients affected are women. Actually, most affected women are postmenopausal, suggesting that ovary hormones could play a protective role in the hair follicles of the affected zones [8,9]. According to previous reports, FFA onset occurs at a postmenopausal age in 83% to 95% of women [8,10,11,12]. Early menopause could be a triggering factor causing a higher risk of developing this disease or premature cases [8]. In addition, FFA is often associated with autoimmune disorders, the most frequent being one hypothyroidism, observed in between 8 and 44% of patients according to different reports [8,10,11]. Other common autoimmune associations are with systemic lupus erythematosus, vitiligo, psoriasis or different forms of lichen planus, especially LPP [8,10,11,13,14]. Interestingly, some genetic factors have been proposed as well, as there are a few reports describing cases of FFA in genetically related individuals [15,16]. Porriño et al. described a series of 20 patients with FFA from nine different families, noticing an early onset in daughters of mothers with this condition [16]. Lastly, some external factors have been linked to the development of FFA, such as surgical procedures of the scalp (e.g., hair transplants) [17,18] or the use of sunscreen [19].

The clinical course of FFA is variable but usually entails an insidious progression which may stabilize after several years [11]. In relation to the treatment, there are no standard treatments for these patients, and the main aim of clinicians is to alleviate symptoms, appease inflammatory signs and stabilize the progression, since hair regrowth is not possible in already alopecic areas. Some of the treatments that have shown certain benefits are corticosteroids (topical, intralesional or systemic), topical calcineurin inhibitors, hydroxychloroquine or 5-alpha-reductase inhibitors (such as finasteride and dutasteride) [8,20]. In a large retrospective study conducted by Vañó et al., a total of 111 patients were treated with 5-alpha-reductase inhibitors, the most effective therapy included in the study, achieving stabilization in 53% of patients and improvement in 47% [8].

Despite its increasing incidence, the pathogenesis of FFA remains unknown and its treatment still represents a challenge. In this context, the aim of this study was to describe the epidemiology, clinical and trichoscopic features, diagnostic findings and comorbidities of a series of 306 patients with FFA.

## 2. Materials and Methods

Studied population:

A retrospective, observational single-center study was conducted in the Hospital General Universitario in Ciudad Real, Spain. In this study, patients with a diagnosis of FFA between 2010 and 2021 were included. All diagnoses were made following well-established diagnostic criteria which included clinical, trichoscopic and even histopathological aspects, as previously described [21]. These diagnostic criteria are summarized in Table 1.

From each patient meeting the criteria described above, epidemiological data (age, gender, postmenopausal onset, age of onset and progression time), clinical presentations (clinical severity, clinical pattern, eyebrow and eyelash alopecia, body hair involvement, association with androgenetic alopecia and presence of facial papules), symptoms (pruritus and trichodynia), trichoscopy (perifollicular erythema and follicular hyperkeratosis), histological confirmation of FFA or LPP and comorbidities (cutaneous and mucosal lichen planus, LPP, hypothyroidism and other autoimmune disorders) were collected and analyzed. The clinical and trichoscopic findings were gathered through clinical histories or retrospectively evaluated using clinical photographs of the patients.

The clinical pattern distribution was classified as pattern I (homogeneous linear frontotemporal recession), pattern II (hair density loss behind a preserved frontal hairline) or pattern III (alopecic band following a preserved frontal hairline) [22], and other unusual patterns were classified as AGA-like (when the recession spares the paramedian frontal hairline), cockade-like (oval patches affecting each temporal region), ophiasis-like (when the alopecic band extends to the occipital hairline) or upsilon-like (when the frontotemporal involvement extends to the parietal scalp, forming a triangle shape) [23,24].

The clinical severity of FFA was assessed by using a five-grade classification measuring the distance between the original frontal hairline and the present one, considering the largest measurement as defining the grade of severity. The proposed grades were as follows: grade I (<1 cm), grade II (1–2.99 cm), grade III (3–4.99 cm), grade IV (5–6.99 cm) and grade V (≥7 cm), also referred as “clown alopecia” [8]. These grades were later grouped as either mild FFA (grades I and II) or severe FFA (grades III, IV and V) for statistical analyses.

The prevalence and standardized incidence were calculated based on the assigned population of the “Gerencia de Atención Integrada” of Ciudad Real, a total of 193,881 persons in the year 2021. Therefore, this represents the potential population attended by our hospital during that year.

Statistical analyses:

Continuous variables were expressed as median and range and categorical variables were expressed as frequencies. A Chi-square test (χ^2^) was employed for categorical variables. A Kolmogorov–Smirnov test was used to verify a normal distribution. A Mann–Whitney U test or a Student’s *t*-test were employed for categorical dichotomous variables. Pearson or Spearman tests were used to assess correlation. The models were controlled for potential confounders based on biological plausibility, published factors and those variables with *p* values < 0.20 in the bivariate analysis. A *p* value ≤ 0.05 was considered statistically significant (CI 95%). Statistical analyses were performed using R software version 4.2.1 (R Project for Statistical Computing).

## 3. Results

Searching by a codified diagnosis of lichen planopilaris and scarring alopecia, we gathered a total of 432 patients. Applying our inclusion and exclusion criteria, we excluded patients with other forms of scarring alopecia or patients with probably incipient FFA which did not fulfill the diagnostic criteria to be classified as such. A total of 306 patients with FFA were included in the study, 296 of them were women (96.7%) and 10 were men (3.3%). The mean age of patients included was 68.3 years old (y/o), ranging from 40 to 98 years old. Among female patients, 246 (84%) were post-menopausal when diagnosed, whereas 47 (16%) had not reached menopause yet. The mean age of FFA onset was 59.5, ranging from 30 to 88 years. The mean time of progression before clinical diagnosis was 8.8 years (Table 2).

**Figure 2:**
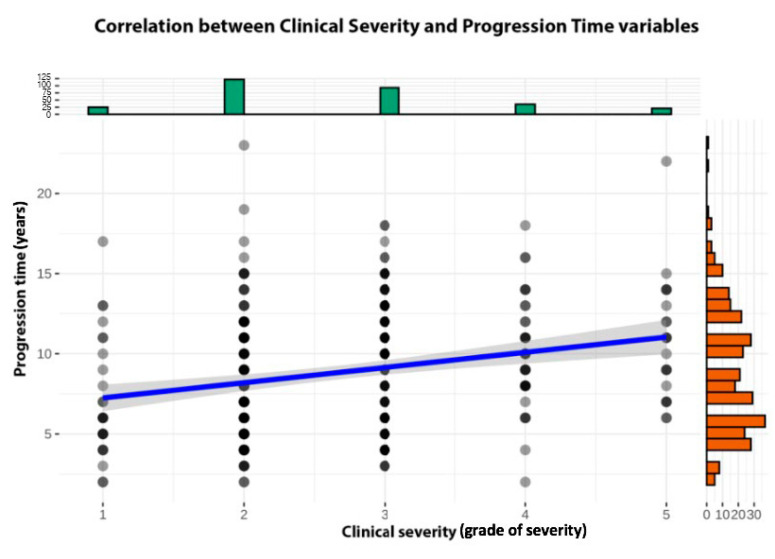
Graphic representing the Spearman rank correlation rho between severity (grades I to V; x axis) and progression time (in years, y axis). Each dot represents a data point from each patient, and columns represent the density of these data. The slope of the line indicates a positive, statistically significant and medium (rho = 0.26, S = 3.18 × 10^6^, *p* < 0.001) correlation between both variables.

Regarding the FFA severity of the disease, 25 patients (8.4%) had grade I, 122 patients (41.2%) had grade II, 93 patients (31.4%) had grade III, 35 patients (11.8%) had grade IV and 21 had grade V (7.1%) alopecia. There was an even distribution between mild FFA (147 patients, 49.7%) and severe FFA (149 patients, 50.3%). We found a correlation between the severity of the disease and the time of progression since onset. This correlation was positive, statistically significant and medium (rho = 0.26, *p* = 0.001) (Figure 2).

Concerning the clinical distribution pattern, the most frequent pattern was pattern I, with 184 patients (62.2%), followed by 48 patients (16.2%) with pattern II and 22 (7.4%) with pattern III. As regards unusual patterns, 14 patients (4.7%) presented an AGA-like pattern, 7 (2.4%) a cockade-like pattern, 17 (5.7%) an ophiasis-like pattern and 4 (1.4%) an upsilon pattern. In addition, there was eyebrow alopecia in 277 patients (90.5%), eyelash alopecia in 35 patients (11.5%) and body hair involvement in 84 patients (38%) (Table 3). Performing a multivariate analysis, we found eyebrow alopecia was associated with milder forms of FFA (OR = 0.38, CI 0.15–0.98, *p* = 0.04). In contrast, we did not observe a statistically significant association between the FFA severity and the presence of facial papules (OR = 1.35, CI 0.69–2.63, *p* = 0.37) or hair body alopecia (OR = 0.62, CI 0.35–1.10, *p* = 0.1).

A concomitant and variable grade of androgenetic alopecia was seen in 82 patients (27.2%). Facial papules were present in 65 patients (21.5%). The hair pull test was positive in 54 patients (17.6%).

The most related symptom was pruritus, which affected 162 patients (63.3%), while only 16 patients (6.2%) suffered from trichodynia. Furthermore, inflammatory trichoscopic signs include perifollicular erythema, which was seen in 206 patients (67.3%), and follicular hyperkeratosis, seen in 188 patients (61.4%). The severity of the disease was not associated with the trichoscopic presence of perifollicular erythema (*p* = 0.09) or follicular hyperkeratosis (*p* = 0.07). Therefore, the clinical progression and FFA severity seem not to be related to the presence of inflammatory trichoscopic signs.

When the clinical findings were insufficient to establish an FFA diagnosis, histopathological analyses were performed. In this sense, histology was conducted in samples from the frontotemporal hairline in a total of 56 patients (18.3%). The main histological findings were the presence of lymphocytic infiltrate follicular fibrosis and hair destruction.

Concomitant signs of classic LPP were found in 30 patients (9.8%), and previous history of cutaneous, oral and genital lichen planus was uncommon (5, 10 and 3 patients: 1.6%, 3.3% and 1%, respectively). A total of 123 patients (40.2%) had other associated autoimmune disorders, the most frequent was hypothyroidism (70 patients, 22.9%), followed by alopecia areata in 12 patients (3.9%), psoriasis in 8 patients (2.6%) and vitiligo in 5 patients (1.6%).

According to our data, the estimated prevalence for our population was 0.159%. The standardized incidence for FFA in 2018 in our population was 15.47 new cases per 100,000 inhabitants.

## 4. Discussion

FFA is a primary lymphocytic scarring alopecia, initially described as a variant of LPP with a distinctive clinical presentation. It is still a discussed topic whether FFA represents its own entity or if it is a variant of LPP [6,25,26]. The clinical presentation of LPP consists of one or a few areas of scarring alopecia, usually involving parietal areas and the vertex [4]. The histopathological findings are similar in both entities, showing a lymphocytic infiltrate around the infundibulum and isthmus, follicular fibrosis and, lastly, hair destruction [1]. The association between AFF and other forms of lichen planus is infrequent, coinciding with our results, whereas the association between LPP and other forms of lichen planus is much more common [7].

In New York City (U.S.A), the overall crude prevalence has been recently estimated to be 0.015%, with an incidence of 5.41 cases per 100,000 inhabitants per year [27,28]; these data are notably lower than our results. Unfortunately, there are no data regarding the incidence or prevalence of FFA in our environment and thus future multicentric studies are still needed to determine these key aspects of FFA.

Initially, FFA was described exclusively in postmenopausal women, which remains the most affected group, although it has also been described in premenopausal women and men. The percentage of premenopausal women in different series ranges from 5% to 17% [8,10,11,12], with our results being closer to the higher percentage. The mean age of onset in our cohort is in line with the range reported in the literature, at between 56 and 63 years old [8,10,12], when menopause has been established in most women. Nevertheless, premenopausal cases seem to be increasing as well; as described here, a noticeable number of women (47 patients, 16%) in our study were of childbearing age, higher than older reports [6,11]. Therefore, FFA may affect younger patients, where the youngest one reported, to our knowledge, is a 15-year-old female in a series of 490 cases studied by Kanti et al. [12]. In addition, a previous study described a higher incidence of early menopause, at 14% among female patients, while the incidence in the general population is estimated at 6% [8]. This increment could be related to a higher rate of hysterectomies performed in this group of patients.

For male patients, the reported mean age of onset ranges from 46 to 54 years [29], slightly earlier than in our cohort (59.3 years) and only 10 of our 306 patients (3.3%) were male. Eyebrow, sideburn and beard alopecia were reported in up to 94.9%, 89.7% and 74.4%, respectively, in the largest male patient series to date [30]. This information slightly differs from our results (80%, 70% and 80%, respectively). Due to the overlap with the much more frequent AGA in men, FFA in males is surely underdiagnosed, who may only look for medical attention when there is sideburn, facial hair, or eyebrow alopecia.

As mentioned before, many autoimmune diseases have been associated with FFA, suggesting the involvement of an autoimmune mechanism in the pathogenesis of this condition. In fact, up to 30% of patients with FFA suffer from another autoimmune condition, the most frequent being thyroid diseases, specifically hypothyroidism (8–44%) [8,11,31]. Other reported associations in line with our findings are psoriasis (7.4%), alopecia areata (0.6–1.7%), vitiligo (0.6–5.6%) or systemic lupus erythematosus (3.4%) [4,8,13,32,33]. Classical LPP is the form of lichen planus most usually seen in patients with FFA, present in up to 25% of patients. The remaining forms of lichen planus are rarely described in association with FFA, which is similar to our report [7]. However, some authors have found higher incidences of lichen sclerosus compared to general population, such as Grassi et al., who reported that 10.6% of their 119 patient cohort suffered from both FFA and lichen sclerosus [34].

Clinically, the predominant symptom of FFA is pruritus in the frontotemporal hairline, which can be found in up to 50% of patients but was slightly higher in our cohort (63.3%), followed by trichodynia [5,8]. According to published information, eyebrow alopecia is seen in 63% to 83% of patients [3,8,11], differing from our results, as this condition was more frequent within our studied population (90.5%). It could be related to the fact that eyebrow alopecia was used as a major criterion for inclusion in our study. Moreover, it may also precede hair line recession in up to a third of the cases [35,36]. Therefore, in the presence of isolated eyebrow alopecia, a skin biopsy of this area may lead to an early diagnosis of FFA. This could serve as a start for early treatment on these patients in order to avoid or delay the disfiguring hair loss of the scalp. Clinical presentation in the form of eyebrow alopecia has been associated with milder forms of disease, which is supported by our outcomes [37]. Eyelash alopecia is less frequently seen, affecting between 3 and 14% of patients with FFA [8,11]. Moreover, the percentage of patients with body hair loss is highly variable in different reports (ranging from 22 to 77%). This high variation could be explained by a confusion with body hair loss due to hormonal changes in postmenopausal women instead of by FFA [11,31]. Facial papules have also been observed in patients with FFA and they could indicate that vellus facial hairs are affected. Facial papules are unspecific for FFA but they can be considered a usual sign in patients with FFA as they may affect between 6% and 37% of patients [38,39]. In our population, the prevalence was within this range, affecting 21.5% of patients.

**Figure 3:**
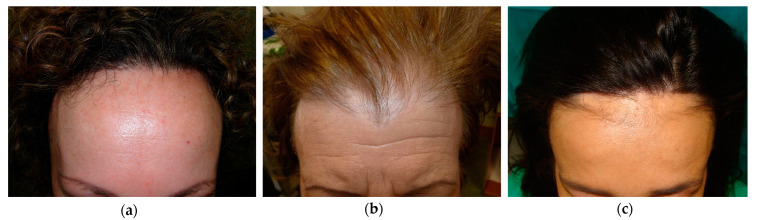
Usual patterns of presentation of AFF: (**a**) Pattern I: a homogeneous symmetric and linear recession of the frontotemporal hairline; (**b**) Pattern II: hair density loss affecting the frontotemporal scalp, sparring the original hairline and leading to diffuse alopecia; (**c**) Pattern III: alopecic band affecting the frontotemporal area, sparring the primitive hairline.

FFA can be classified as various patterns regarding the clinical affectation of the hairline. Some of these patterns suggest a different clinical course and prognosis [22]. Pattern I or a “linear pattern” has been reported to be the most frequent one, approaching 50% of total patients, with an intermediate prognosis considering disease progression. Pattern II or a “diffuse pattern” is the second most usual pattern (around 45%), and appears to have the worst prognosis, regarding a more frequent progression despite treatment and a higher severity at the moment of diagnosis. Our cohort showed a greater number of patients with pattern I (62.2%) and a significantly lower amount with pattern II (16.2%). The latter can be overlapped or misdiagnosed as AGA or fibrosing alopecia in pattern distribution due to the diffuse alopecia in this pattern. Pattern III or a “pseudo fringe-sign pattern” is the less common of these three (6.2%) and seems to have the best prognosis, with the majority of patients remaining stable with appropriate treatment (Figure 3). This pattern often spares eyebrow and eyelash hair [40]. Other unusual patterns have been described, accounting for 18.4% of patients in some studies, similar to our results (14.2%). These patterns are AGA-like patterns, ophiasis-like patterns, cockade-like patterns and upsilon patterns, with former two being the most common [23,24].

**Figure 4:**
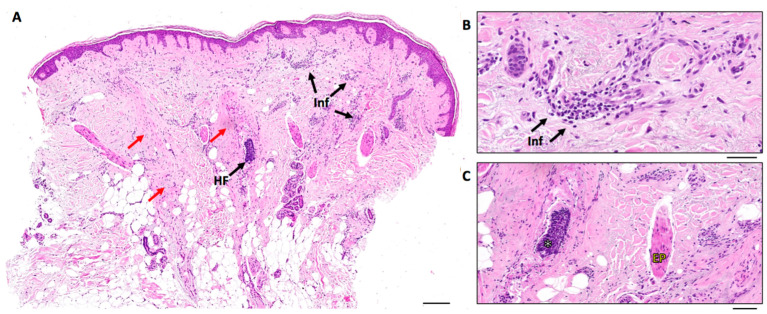
Histological features of patients affected by FFA. Images correspond to a 66-year-old woman with five years of FFA evolution. Image (**A**) shows a skin biopsy stained with hematoxylin–eosin at low magnification, where it is possible to observe an absence of hair follicles (HF), some HF remnants (HF and black arrow), cicatricial changes within the dermis (red arrows) and inflammatory elements (Inf and arrows). Image (**B**) shows the inflammatory elements within the dermis at higher magnification (Inf and arrows). Image (**C**) shows the remnants of HF (white asterisk) and the erector pili smooth muscle (EP). Scale bar: 200 µm (**A**), 50 µm (**B**,**C**).

A histological study of samples from the recession line was characterized by a lymphocytic infiltrate in a lichenoid pattern, involving the upper follicle and accompanied by concentric perifollicular lamellar fibrosis (Figure 4) [1]. When maintained over time, there is a decrease in the number of hair follicles, which are substituted by fibrous cicatricial tracts. Initially, the disease equally affects terminal and vellus hairs, although in later stages, the involvement is focused on terminal hair follicles [36,41]. The disappearance of sebaceous glands seems to be the earliest histological sign in patients with FFA, and its preservation in eyebrows might be correlated with the reversibility of the alopecia [37,42]. Despite the fact that the hormonal influence is thought to play an important role in the pathogenesis of this entity, no alterations in hormonal receptors have been described yet.

Whether AFF is a variant of LPP or a distinctive entity is yet to be elucidated. Both pathologies share primary histological findings, although clinically LPP often produces alopecic plaques in the vertex and parietal scalp rather than a homogeneous recession on the frontotemporal hairline [4,7]. Histologically, the inflammatory infiltrate is denser in LPP compared to AFF and the damage to the basal layer seems to be more noticeable in the former [7,43]. Moreover, a lymphocytic perivascular infiltrate is more commonly seen in LPP, which also tends to affect the interfollicular epidermis, usually spared in FFA [7]. In addition, LPP is associated with lichen planus at other localizations more often than AFF, such as cutaneous, mucosal or pilaris [7]. In this line, it is still necessary to design new prospective clinical and/or molecular studies focused on the develop new quantitative diagnostic criteria of this frequent pathological condition.

Regarding treatments for FFA, there are no standardized guidelines for these patients, since its etiopathogenesis remains to be clarified. The goals of treatment of this condition are to reduce clinical signs of inflammation, stop the progression of alopecia and appease clinical symptoms. Potent topical corticosteroids and calcineurin inhibitors reduce the inflammation signs and ameliorate symptoms, but do not seem to interfere with the progression of alopecia [11,44].

Among systemic therapies, 5-alpha reductase inhibitors appear to be the most effective treatment, as they seem to achieve the highest stabilization rates [8,44,45]. Oral dutasteride is a well-tolerated treatment, which offers a greater stabilization rate compared to other systemic therapies [46]. Hydroxychloroquine is another systemic treatment commonly used, with similar responses rates to those of 5-alfa reductase inhibitors, but mainly partial responses [10]. Hair transplants should be offered with caution to these patients, as the graft survival rate may be lower than expected even after years without clinical activity of FFA [47,48].

Our study has some limitations, the primary one being the observational and retrospective design, as it limits the collection of representative data. In addition, it is still necessary to evaluate the potential efficacy of the diverse treatments which are in used and their correlation with FFA progression. Despite these limitations, the single-center nature of the study provides a highly homogeneous assessment from the investigators involved and these results could be considered representative of this locality.

In conclusion, this is probably the largest retrospective single-center study analyzing a cohort of FFA patients reported in the literature. FFA is a scarring type of alopecia affecting mainly postmenopausal women, inducing a recession of the frontotemporal hairline. We found a slightly increased number of premenopausal women in our study compared to previous ones (16%). Its pathogenesis still remains unknown, although there is evidence that hormonal factors, genetic susceptibility, autoimmunity and some exogen factors may play an important role. The grade of severity of the alopecia is correlated with the time of progression of the disease. We found eyebrow alopecia was associated with milder forms of FFA, although we did not find an association between the grade of severity and the presence of facial papules or hair body alopecia, as other studies suggest. In our study, the presence or absence of inflammatory trichoscopic signs did not seem to relate to the progression and therefore the severity of FFA, which might change our therapeutic approach for this entity. In addition, to our knowledge, this is the first study conducted in this particular population that shows the incidence, prevalence and main clinical features associated with this entity. Moreover, it is important to conduct more retrospective or prospective clinical studies to develop new quantitative diagnostic criteria to establish and grade the diagnosis of FFA. Finally, histological and molecular studies are still needed to elucidate the biological processes that could be involved in the genesis, progression or response to new treatments of this pathological condition.

## Figures and Tables

**Table 1:** Diagnostic criteria for FFA ^1^.

Major Criteria	Minor Criteria
Cicatricial alopecia of the frontal, temporal or frontotemporal scalp on examination, in the absence of follicular keratotic papules on the body	Typical trichoscopic features: perifollicular erythema, follicular hyperkeratosis or both Histopathologic features of cicatricial alopecia in the pattern of FFA and LPP on biopsy
2. Diffuse bilateral eyebrow alopecia	3. Involvement (hair loss or perifollicular erythema) of additional FFA sites: occipital area, facial hair, sideburns or body hair 4. Noninflammatory facial papules

^1^ Diagnosis of FFA requires both major criteria or one major criterion and two minor criteria.

**Table 2:** Demographic similarities in the presentation of FFA between women and men.

	Women (296 Patients, 96.7%)	Men (10 Patients, 3.3%)	Total (306 Patients, 100%)
Mean age, years	68.3	68.1	68.3
Mean age of onset, years	59.5	59.3	59.5
Time of progression, years	8.8	8.8	8.8

**Table 3:** Clinical and trichoscopic findings in premenopausal women, postmenopausal women and men.

	Premenopausal Women (47 Patients, 16%)	Postmenopausal Women (246 Patients, 83.9%)	Men (10 Patients, 3.3%)	Total (306 Patients, 100%)
Eyebrow alopecia	43 (91.4%)	223 (90.6%)	8 (80%)	277 (90.5%)
Eyelash alopecia	4 (8.5%)	30 (12.3%)	1 (10%)	35 (11.5%)
Body hair alopecia	15 (42.8%)	60 (34.6%)	9 (90%)	84 (38%)
Facial papules	17 (36.1%)	47 (19.4%)	1 (10%)	65 (21.5%)
Androgenetic alopecia	4 (8.5%)	67 (27.6%)	9 (90%)	65 (21.5%)
Predominant pattern	I	I	I	I
Median grade of severity	II	II	III	II
Pruritus	29 (69%)	127 (62.8%)	6 (66.6%)	162 (63.3%)
Trichodynia	5 (11.9%)	10 (4.9%)	1 (11.1%)	16 (6.2%)
Perifollicular erythema	27 (57.4%)	171 (69.5%)	7 (70%)	206 (67.3%)
Follicular hyperkeratosis	20 (42.5%)	161 (65.4%)	5 (50%)	188 (61.4%)

## Data Availability

Not applicable.

## Abbreviations

FFA: frontal fibrosing alopecia; LPP: lichen planopilaris; AGA: androgenetic alopecia.
